# Mediating Effects of Social Support and Resilience on the Association between COVID-19-Related Stress and Mental Health in Korean Young Adults

**DOI:** 10.3390/ijerph19116935

**Published:** 2022-06-06

**Authors:** Dabok Noh, Suin Park

**Affiliations:** 1 College of Nursing, Eulji University, Seongnam 13135, Korea; daboknoh@gmail.com; 2 College of Nursing, Kosin University, Busan 49267, Korea

**Keywords:** anxiety, COVID-19-related stress, depression, resilience, social support, young adult

## Abstract

Young adults were vulnerable to mental health issues during the COVID-19 pandemic. However, the underlying pathway by which COVID-19-related stress influences mental health outcomes among young adults remains unclear. This study aimed to examine the mediating effects of social support and resilience between COVID-19-related stress and mental health outcomes. A sample of 1000 Korean young adults was obtained via online survey. Participants completed self-report questionnaires assessing COVID-19-related stress, social support, resilience, depression, and anxiety. Overall, 48.1% and 23.4% of participants were classified as having depression and anxiety states, respectively. Path analysis using AMOS version 26.0 (IBM Corp., Armonk, NY, USA) showed that the direct effects of stress from social distancing difficulties on depression and anxiety were much greater than those of stress from fear of infection and anger toward others. In addition, there were significant indirect effects of social support and resilience in the relationship between stress related to difficulties due to social distancing and mental health outcomes. The mediating roles of social support and resilience suggest that interventions to increase these factors can be effective strategies to reduce the risks of depression and anxiety among young adults suffering from stress related to social distancing difficulties.

## 1. Introduction

The COVID-19 pandemic is a global crisis that has caused life disruption and affected mental health [1]. In line with global regulations, the Korean government established public health regulations that included mandatory quarantines, social distancing, and lockdowns of facilities or businesses to limit the spread of COVID-19 [2]. These regulations contributed to decreased economic activity, increased unemployment, and reduced social support, which, along with the threat of COVID-19 infection, had adverse impacts on mental health [1,2,3].

Young adults, who are in emerging adulthood or late adolescence, experience a stressful transition period that includes instability related to changes in educational and residential status and relationships, finding a new job, and financial hardship [3,4,5]. Young adults also may experience anxiety due to identity issues that are prominent in emerging adulthood. Moreover, some may experience serious mental health problems because their lives are less structured than children, adolescents, or older adults [6]. In this context, young adults should be concerned about their mental health vulnerability which could be exacerbated by COVID-19-related stress. Therefore, it is important to examine the mental health status among young adults and factors affecting their mental health during the COVID-19 pandemic.

### 1.1. Psychological Impact of COVID-19 Related Stress among Young Adults

Previous studies have shown that young adults are vulnerable to COVID-19 pandemic stress due to a loss of routine, lack of social contact, and work and financial concerns; this stress is linked to an increased risk of impaired mental health outcomes [7,8,9]. Indeed, young adults aged 18–24 years have experienced higher levels of depression and anxiety during the COVID-19 pandemic than adults aged 25 years or more [10]. A previous longitudinal cohort study has reported that young adults showed higher perceived stress and anger during the COVID-19 pandemic than before the pandemic. Additionally, economic and psychosocial stressors influenced their emotional distress during the pandemic [3]. Moreover, psychological distress due to COVID-19 is associated with mental health problems related to Internet and Instagram addiction among young adults [11].

COVID-19-related stress can be categorized into fear of infection, difficulties due to social distancing, and anger toward others who do not comply with quarantine guidelines [12]. First, fear of being infected or infecting others is one of the stressors during COVID-19 pandemic, and impact of fear of COVID-19 infection on mental health including depression and anxiety has been reported [13,14]. Second, mandatory quarantines and social distancing leads to a loss of routine activities and reduced in-person contact with others, which in turn causes feelings of isolation, boredom, and loneliness [15]. Social isolation and loneliness have increased depression and suicide rates during the pandemic [16,17]. Among young adults, social distancing measures have resulted in increased feeling of loneliness when compared with older adults [18]. Since emerging adults experience numerous changes in their relationships, social distancing may restrict the successful development of relationships, which could negatively affect their mental health [6]. Third, individuals’ personal efforts to reduce the spread of COVID-19 are more likely to lead to anger toward others’ behaviors that increase the risk of transmission [12]. A previous study has reported positive correlations among COVID-19-related anger, depression, and anxiety [19]. Taken together, these studies highlight that COVID-19-related stress influences mental health outcomes including depression and anxiety among young adults.

### 1.2. Relationships among COVID-19-Related Stress, Social Support, Resilience, and Mental Health

According to the stress-buffering model, social support can be a protective factor from the detrimental effects of stress on mental health outcomes during times of adversity [20]. Social support is defined as social resources that can be obtained from both formal and informal helping relationships [21]. Previous findings have demonstrated a buffering role of social support for mental health from traumatic experiences or disasters [22,23]. Additionally, previous studies during the COVID-19 pandemic have reported an association between social support and depression [24].

Psychological resilience is an individual internal resource that refers to the ability to adapt effectively in the face of adversity, such as during stress, potentially traumatic events, or losses; it helps individuals to overcome and grow through adversity [25,26]. Previous studies have reported an association between resilience and mental health outcomes, including depression, anxiety, and post-traumatic symptoms [27,28]. A study of young adults during the COVID-19 pandemic has shown that high resilience is associated with low depression and anxiety [24].

Social support and resilience may have sequential mediating roles against the impact of COVID-19-related stress on depression and anxiety. When stressors occur, social support as an external factor can help the individual resiliency process toward adaptation [29,30]. Social support may play an important role in enhancing resilience [31,32]. Furthermore, there is a mediating effect of resilience on the association between social support and mental health outcomes among health care workers during the pandemic [33]. The mediation effect of resilience on the relationship between social support and subjective well-being has been reported among individuals with spinal cord injuries [30]. Moreover, a sequential mediating effect of social support and resilience on the association between COVID-19-related work stress and mental health outcomes, including depression and anxiety, has been reported [34]. Therefore, the association between COVID-19-related stress and mental health outcomes could be mediated by social support and resilience among young adults.

### 1.3. The Current Study

**Figure 1:**
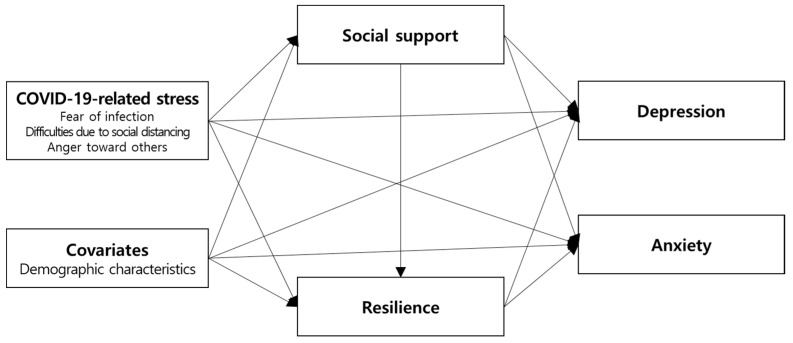
A hypothetical model of the study.

The theoretical concepts in this study were derived from the resilience framework [29]. According to this framework, an interaction of the individual with the external factors is activated in the face of stressors or challenges. Next, the resilience process occurs sequentially via internal resilience factors. Finally, mental health outcomes of adaptation appear [29]. We applied the framework as follows: COVID-19-related stress was considered as the stressor or challenge, social support was considered as an external factor, resilience was considered as an internal factor, and levels of depression and anxiety were considered as mental health outcomes. Figure 1 shows our hypothesis model, in which COVID-19-related stress influences depression and anxiety, and this relationship is mediated by social support and resilience.

In order to clearly identify the relationship among predictors, mediators, and outcome variables, confounding factors such as demographic variables should be considered. It has been reported that demographic factors influence depression and anxiety. During the pandemic, gender, residence status, living situation, and financial resource were significantly associated with depression among university students [13]. Specifically, increased depression and anxiety were associated with females, a younger age [1,10], and lower income [1]. Previous studies that have examined the predictors of depression and anxiety during the COVID-19 pandemic show models that are adjusted for gender [24,35,36], age [24,35], education level [35,36], religion [36], cohabiting family [36], income [24], parental occupational status [35], and employment status [36] as covariates. Therefore, we included demographic factors such as gender, age, education level, religion, living with spouse, residential area, and household economic status, which could affect depression and anxiety in our hypothesized model.

Previous studies of young adults facing the COVID-19 pandemic have examined the impact of COVID-19 on mental health [10,37] and associated factors [24]. However, few studies have examined the sequential mediating roles of social support and resilience on the association between COVID-19-related stress and mental health outcomes among young adults. Young adults are more vulnerable to the impact of the COVID-19 pandemic on mental health [10]; therefore, understanding the underlying pathway by which COVID-19-related stress influences mental health outcomes among young adults is important.

This study aimed to (a) examine the differences in social support, resilience, depression, and anxiety according to demographic characteristics; (b) identify levels of COVID-19-related stress, social support, resilience, depression, and anxiety among young adults; and (c) examine the mediating effects of social support and resilience on the relationship between COVID-19-related stress and mental health outcomes (depression and anxiety). In particular, these main hypotheses were tested using a path analysis:

**Hypothesis** **1** **(H1).** *Fear of infection, difficulties due to social distancing, and anger toward others directly influence depression and anxiety, respectively;*

**Hypothesis** **2** **(H2).** *Social support directly influences depression and anxiety;*

**Hypothesis** **3** **(H3).** *Resilience directly influences depression and anxiety;*

**Hypothesis** **4** **(H4).** *Social support indirectly influences depression and anxiety through mediating effect of resilience;*

**Hypothesis** **5** **(H5).** *Fear of infection, difficulties due to social distancing, and anger toward others indirectly influence depression and anxiety, respectively, through the sequential mediating effects of social support and resilience.*

## 2. Materials and Methods

### 2.1. Study Design and Participants

This study used a descriptive, cross-sectional study design. The sample size was 1000 participants. The inclusion criteria were: (1) Korean young adults aged 19–24 years (this age range was followed the definition of young adult in Medical Subject Headings [38]); (2) individuals who understood the purpose of the research and voluntarily consented to participate in the research; and (3) individuals who were capable of using the internet and e-mail. Those who did not consent to participate in this study were excluded.

### 2.2. Measures

#### 2.2.1. COVID-19-Related Stress

COVID-19-related stress was measured using the COVID-19 Stress Scale for Korean People (CSSK) developed by Kim et al. [12]. The CSSK is a self-report questionnaire designed to measure COVID-19-related stress among Korean adults. The scale comprises three subscales: ‘fear of infection’ reflecting concerns regarding COVID-19 infection (9 items); ‘difficulties due to social distancing’ reflecting frustration, helplessness, depression, and financial concern felt by not being able to perform everyday life or social life activities properly due to social distancing (6 items); and ‘anger toward others’ reflecting anger toward individuals or groups who do not comply with quarantine guidelines (6 items) [12]. An example of the items of ‘fear of infection’ is “I am worried about becoming seriously ill because of COVID-19.” An example of the items of ‘difficulties due to social distancing’ is “It is distressing that I am not able to meet family members or friends as often due to COVID-19.” An example of the items of ‘anger toward others’ is “I am angry with people who do not follow quarantine orders” [12]. COVID-19-related stress was analyzed by dividing it into three observational variables because the differences in the concepts of the three subscales were clear. A five-point Likert scale ranging from 0 (strongly disagree) to 4 (strongly agree) was used. Higher scores indicated a higher level of COVID-19-related stress. In a prior study, Cronbach’s α for ‘fear of infection,’ ‘difficulties due to social distancing,’ and ‘anger toward others’ was 0.93, 0.81 and 0.89, respectively [12]. In this study, Cronbach’s α was 0.91, 0.82 and 0.87, respectively.

#### 2.2.2. Social Support

We used the Korean version of the Social Provision Scale (SPS) developed by Cutrona and Russell [39] and translated into Korean by Yoo and Lee [40] to assess each participant’s perceived social support. The scale comprises 24 items scored on a four-point Likert scale ranging from 1 (strongly disagree) to 4 (strongly agree), among which the 12 negatively worded items were reverse coded. An example of the questionnaire items is “There are people I can depend on to help me if I really need it.” Higher scores indicate greater perceived social support, with scores ranging from 24 to 96. Cronbach’s α was 0.92 in a prior study [39] and 0.81 in this study.

#### 2.2.3. Resilience

Resilience was measured using the Korean version of the Connor–Davidson Resilience Scale (K-CD-RISC) developed by Connor and Davidson [25] and standardized by Baek et al. [41]. The scale consists of 25 self-reported items scored on a five-point Likert scale ranging from 0 (not at all) to 4 (almost always). An example of the questionnaire items is “able to adapt to change.” Higher scores indicate greater resilience, with scores ranging from 0 to 100. Cronbach’s α was 0.89 in a prior study [25] and 0.94 in this study.

#### 2.2.4. Depression

Participants’ depression was assessed using the Korean version of the Center for Epidemiologic Studies Depression Scale-Revised (CESD-R). This is a tool that Lee et al. [42] standardized from the CESD-R [43] into the Korean version. This scale is in the public domain and is freely available for research. It consists of 20 self-reported items on a five-point Likert scale ranging from 0 (not at all or less than one day last week) to 4 (nearly every day for two weeks). An example of the questionnaire items is “I could not shake off the blues.” Higher scores indicate a higher level of depression, with scores ranging from 0 to 80. The Korean version of the CESD-R predicts depression with 96% sensitivity and 96% specificity when the cutoff value is set to 13 or higher [42]; therefore, this study set a cutoff score of 13 or higher for the screening of depressive symptoms. Cronbach’s α coefficient of the Korean version of the CESD-R was 0.98 in a prior study [42] and 0.95 in this study.

#### 2.2.5. Anxiety

Anxiety was measured using the Korean version of the Generalized Anxiety Disorder-7 (GAD-7) questionnaire developed by Spitzer et al. [44]. The Korean version of the GAD-7 questionnaire is freely available in research and can be downloaded from the public domain. The scale consists of 7 self-reported items that assess how often individuals have been bothered by the problems (e.g., feeling nervous, anxious, or on edge) over the prior 2 weeks. Each item is scored on four-point Likert scale ranging from 0 (not at all) to 3 (nearly every day). Higher scores indicate a higher level of anxiety, with scores ranging from 0 to 21. The Korean version of the GAD-7 questionnaire predicts a generalized anxiety disorder with 81% sensitivity and 85% specificity when the cutoff point is set to 8 or higher [45]. Therefore, we also set the cutoff point of the GAD-7 questionnaire to 8 or higher. Cronbach’s α coefficient of the Korean version of the GAD-7 questionnaire was 0.93 in both a previous study [45] and this study.

#### 2.2.6. General Characteristics

We collected gender, age, education level, religion, living with spouse, residential area, and household economic status.

### 2.3. Data Collection

Ethical approval of this study was obtained from the institutional review board of the author’s institution (No: EU21-060). Data were collected from 1 September 2021 to 7 September 2021 using an online survey. The participants were recruited by quota sampling stratified by gender from registrants with the online survey agency, which has its own panel of approximately 700,000 people in Korea. A total of 1000 questionnaires were collected, and all of them were included in the analysis. Participants completed online informed consent.

### 2.4. Data Analyses

The data were analyzed using SPSS version 26.0 and AMOS version 26.0 (IBM Corp., Armonk, NY, USA). Descriptive statistics of demographic characteristics, COVID-19-related stress, social support, resilience, depression, and anxiety were performed. We used bivariate analyses, such as the *t*-test, ANOVA with post hoc Scheffe’s test, and Pearson’s correlations, to identify differences in social support, resilience, depression, and anxiety according to demographic characteristics.

To evaluate the proposed hypothetical model, we used path analysis with AMOS using maximum standard likelihood estimation. In the path diagram, the three subscales of COVID-19-related stress were the independent variables, social support and resilience were the mediating variables, and depression and anxiety were the outcome variables. The demographic variables that were found to be statistically significantly related to the mediating and outcome variables in the prior bivariate analyses were adjusted by considering them as covariates. After fitting the model for all possible path coefficients of the hypothetical model, statistically nonsignificant path coefficients in the output path diagram of hypothetical model were removed, and the modified model was refitted. There are consistent mediation models in which direct effects and indirect effects have same sign, whereas the mediation models in which direct effects and indirect effects have opposing signs are interpreted as inconsistent mediation models [46].

## 3. Results

### 3.1. Social Support, Resilience, Depression, and Anxiety According to Demographic Characteristics

The descriptive statistics of demographic characteristics and of social support, resilience, depression and anxiety according to demographic characteristics are presented in Table 1. Slightly over half of the participants were male (52.1%). Their ages ranged from 19 to 24 years, with a mean age of 21.63 years (SD = 1.69). Regarding education level, college students made up the largest proportion of the participants (64.7%), followed by college graduates (21.3%) and those who had graduated high school or less (14.0%). Most of the respondents had no religion (70.2%). Almost all of the participants had no spouse with whom they lived (98.9%). More than half of the participants lived in a big city (53.7%), while some lived in a small- or medium-sized city (35.8%) or in a rural area (10.5%). The portion of the participants who perceived their household economic status as middle was 38.2%, while 31.6% and 30.2% perceived their household economic status as low and high, respectively.

Females showed significantly higher social support (t = −2.10, *p =* 0.036), depression (t = −2.68, *p* = 0.007) and anxiety (t = −4.44, *p* < 0.001) than males, while they showed significantly lower resilience (t = 4.84, *p* < 0.001). Participants who graduated high school or less had significantly lower social support than college students (F = 3.46, *p =* 0.032). College graduates had significantly higher depression (F = 5.77, *p =* 0.003) and anxiety scores (F = 4.76, *p* = 0.009) than college students. Participants with a religion had higher resilience than participants without a religion (t = −3.00, *p* = 0.003). Participants living in a big city had higher resilience than those living in a small or medium-sized city (F = 3.88, *p* = 0.021). Participants who responded that their household economic status was low had significantly lower social support scores than those who responded that their household economic status was middle or high (F = 11.50, *p* < 0.001). Participants with a high household economic status had significantly higher resilience scores than those with a middle or low household economic status (F = 19.08, *p* < 0.001). In addition, participants with a low household economic status had higher depression (F = 5.13, *p* = 0.006) and anxiety scores (F = 3.12, *p* = 0.044) than those with a high household economic status.

### 3.2. Levels of COVID-19-Related Stress, Social Support, Resilience, Depression, and Anxiety

The participants had a mean COVID-19-related stress score of 57.33 (SD = 13.08), and the average scores of fear for infection, difficulties due to social distancing, and anger toward others were 24.15 (SD = 7.11), 14.10 (SD = 4.84), and 19.09 (SD = 4.31), respectively. The mean perceived social support score was 70.72 (SD = 10.85), and the mean resilience score was 60.43 (SD = 15.73). The participants had a mean depression score of 17.59 (SD = 16.97) and an anxiety score of 4.56 (SD = 5.14). The proportion of participants with depression was 48.1% based on a cutoff score of 13 on the CESD-R. The proportion of participants with anxiety was 23.4% based on a cutoff score of 8 on the GAD-7 questionnaire (Table 2).

### 3.3. Associations among COVID-19-Related Stress, Social Support, Resilience, Depression, and Anxiety

**Figure 2:**
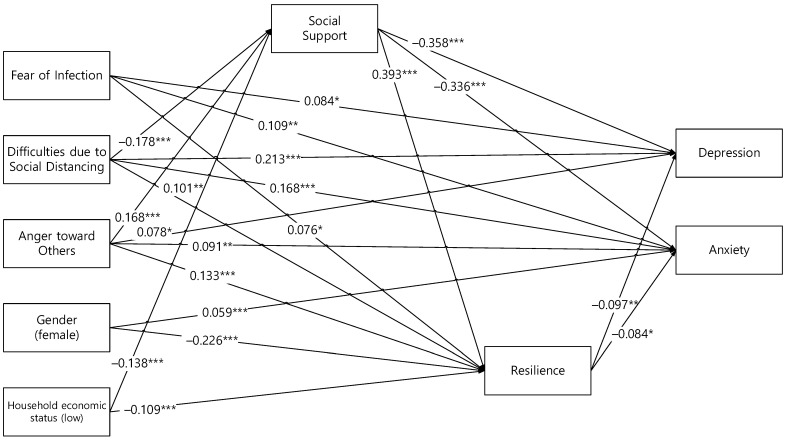
Final path diagram of depression and anxiety among young adults. * *p* < 0.05; ** *p* < 0.01; *** *p* < 0.001. Standardized coefficients are presented.

In our prior bivariate analyses, gender and household economic status were the only demographic variables that were significantly associated with social support, resilience, depression, and anxiety. Therefore, we included these variables as covariates. We constructed the final path diagram of the modified model (Figure 2). The final modified model yielded a good fit for the data (CMIN/df = 1.304, GFI = 0.999, CFI = 0.999, TLI = 0.996, SRMR = 0.010, and RMSEA = 0.017). Table 3 shows the direct, indirect, and total effects of the final modified model.

#### 3.3.1. Path Analysis on the Determinants of Depression

Although the direct effect of gender (female) on depression was not significant, the indirect effect of gender (female) on depression through resilience was significantly positive (β = 0.022, *p* = 0.008). In addition, although the direct effect of household economic status (low) on depression was not significant, the indirect effect of household economic status (low) on depression through social support and resilience was significantly positive (β = 0.065, *p* = 0.001).

The direct effect of fear of infection on depression was significantly positive (β = 0.084, *p* = 0.015); however, the indirect effect of fear of infection on depression through resilience was significantly negative (β = −0.007, *p* = 0.027). Although the total effect of fear of infection on depression was significantly positive (β = 0.076, *p* = 0.028), there was inconsistent mediation because the direct and indirect effects had opposite signs.

The direct effect of difficulties due to social distancing on depression was significantly positive (β = 0.213, *p* = 0.001), and the indirect effect of difficulties due to social distancing on depression through social support and resilience was also significantly positive (β = 0.061, *p* = 0.001). Therefore, the total effect of difficulties due to social distancing on depression was significantly positive (β = 0.274, *p* = 0.001). This showed a consistent mediation model and significant multiple mediator effect of social support and resilience.

The direct effect of anger toward others on depression was significantly positive (β = 0.078, *p* = 0.020); however, the indirect effect of anger toward others on depression through social support and resilience was significantly negative (β = −0.079, *p* = 0.001). The total effect between anger toward others and depression was nonsignificant, and there was inconsistent mediation.

Social support influenced depression directly (β = −0.358, *p* = 0.001) and indirectly through resilience (β = −0.038, *p* = 0.008). Resilience influenced depression directly (β = −0.097, *p* = 0.008). The overall model explained 23.8% of the total variance in depression.

#### 3.3.2. Path Analysis on the Determinants of Anxiety

The direct effect of gender (female) on anxiety was significantly positive (β = 0.059, *p* = 0.001), and the indirect effect of gender (female) on anxiety through resilience was also significantly positive (β = 0.019, *p* = 0.025). Therefore, the total effect of gender (female) on anxiety was significantly positive (β = 0.077, *p* = 0.001). In addition, although the direct effect of household economic status (low) on anxiety was not significant, the indirect effect of household economic status (low) on anxiety through social support and resilience was significantly positive (β = 0.060, *p* = 0.001).

The direct effect of fear of infection on anxiety was significantly positive (β = 0.109, *p* = 0.001); however, the indirect effect of fear of infection on anxiety through resilience was significantly negative (β = −0.006, *p* = 0.039). Although the total effect of fear of infection on anxiety (β = 0.103, *p* = 0.001) was significantly positive, there were inconsistent mediations.

The direct effect of difficulties due to social distancing on anxiety was significantly positive (β = 0.168, *p* = 0.001), and the indirect effect of difficulties due to social distancing on anxiety through social support and resilience was also significantly positive (β = 0.057, *p* = 0.001). The total effect of difficulties due to social distancing on anxiety was significantly positive (β = 0.225, *p* = 0.001). There was a consistent mediation model and a significant multiple mediator effect of social support and resilience.

The direct effect of anger toward others on anxiety was significantly positive (β = 0.091, *p* = 0.007); however, the indirect effect of anger toward others on anxiety through social support and resilience was significantly negative (β = −0.073, *p* = 0.001). The total effect between anger toward others and anxiety was nonsignificant, and there was inconsistent mediation.

Social support influenced anxiety directly (β = −0.336, *p* = 0.001) and indirectly through resilience (β = −0.033, *p* = 0.028). Resilience influenced anxiety directly (β = −0.084, *p* = 0.032). The overall model explained 26.1% of the total variance in anxiety.

## 4. Discussion

### 4.1. Mental Health Status among Korean Young Adults during the COVID-19 Pandemic

In this study sample of Korean young adults aged 19–24 years, 48.1% of respondents were classified as being in a depressive state, and 23.4% of respondents were classified as being in an anxious state. These proportions are higher than those reported for depression (30.7%) and anxiety (22.6%) among Korean adults aged 19–60 years in during the COVID-19 pandemic period [36]. Thus, a greater number of young adults than general adults have reported symptoms of depression and anxiety during the COVID-19 pandemic. Consistent with our interpretation, the U.S. Census Bureau data collected during a period (1 September 2021 to 13 September 2021) were similar to ours; 50.0% of adults aged 18–29 reported symptoms of depression or anxiety, which was higher than the 32.1% of all adults aged 18 years or greater who reported symptoms of depression or anxiety [47]. It is alarming that almost half (48.1%) and almost one-fourth (23.4%) of our sample needed depression or anxiety screening, respectively. This highlights an urgent need to develop an effective approach to assess and intervene in mental health issues among young adults.

### 4.2. The Association between Demographic Factors, Social Support, Resilience, Depression, and Anxiety

Our bivariate analyses revealed gender differences in all measured variables. Although female young adults showed higher perceived social support than male young adults, females had significantly lower resilience and higher scores for depression and anxiety than males. These findings are in accordance with those of previous studies reporting that females had significantly higher levels of depression and anxiety than males during the COVID-19 pandemic [10,37]. These gender differences may be explained because although females experienced greater stress and anxiety than males even before the COVID-19 pandemic, the pandemic increased these gender gaps in mental health because of greater stress response to negative events and adversities in females when compared with males [10].

Similar to our results of bivariate analysis that female young adults had significantly lower average resilience in the face of the COVID-19 pandemic than males, a previous study has reported that the average stress management ability in response to the COVID-19 pandemic among female young adults is significantly lower than that among males [37]. In addition, our path analysis found the significant indirect effects of gender (female) on depression and anxiety mediated by resilience but not mediated by social support. Taken together, our findings suggest that female young adults are vulnerable to depression and anxiety because of their low resilience to the adversity of the COVID-19 pandemic, despite having perceived higher social support than males. Therefore, more attention should be paid to female young adults who are vulnerable to effective responses to this crisis period. Resilience may be a protective factor against depression and anxiety among female young adults; therefore, we suggest that resilience enhancement strategies are required for female young adults. Our results revealed that college graduates showed significantly higher levels of depression and anxiety than college students. In addition, young adults with a low household economic status had significantly higher levels of depression and anxiety than those with a high household economic status. A possible explanation for these findings may be the associations with job insecurity, financial concern, and mental health. A previous study has reported that greater job insecurity due to COVID-19, and the resulting financial concerns, are linked to depressive and anxiety symptoms [48]. Further studies are needed to examine how differences between college students and graduates affect differences in their mental health status. In addition, our path analysis showed a significant indirect effect of household economic status on depression and anxiety, which is mediated by social support and resilience. This finding indicates that social support and resilience may serve as protective factors against depression and anxiety among young adults with low household economic status. Therefore, we suggest increased focus on those with low household economic status and providing interventions to enhance social support and resilience for them.

### 4.3. The Association among COVID-19-Related Stress, Social Support, Resilience, Depression, and Anxiety

This study examined the impact of COVID-19-related stress on depression and anxiety in young adults. It evaluated the indirect effect of COVID-19-related stress on depression and anxiety through social support and resilience via path analysis. The results showed that all three subdomains of COVID-19-related stress (fear of infection, difficulties due to social distancing, and anger toward others) influenced depression and anxiety directly, which supported hypothesis H1. Among the three subdomains of COVID-19-related stress, the direct effects of difficulties due to social distancing on depression and anxiety were much greater than other direct effects. Consistent with our findings, a previous study has stated that complying with social distancing guidelines is a possible reason for poorer mental health in young adults than in older adults [49]. In addition, another previous study documented that college students felt more depressed when their desire for social connectedness was not met due to social distancing [10]. Young adults aged 19–24 years are in a period of exploring love, work, and worldviews; they participate in a wider scope of activities, such as productive activities (school and work), and greater formation of romantic relationships than any other age group [4]. Therefore, in young adults, stress from restrictions on activities due to social distancing during the COVID-19 pandemic may have a greater effect on depression and anxiety. By contrast, our results demonstrated that the fear of infection and anger toward others who do not comply with quarantine guidelines had weak direct effects on depression and anxiety. This can be explained by the fact that young adults have fewer physical symptoms from COVID-19 infection than other age groups [49].

This study revealed that social support and resilience, which are mediating variables, influenced depression and anxiety directly, which supported hypotheses H2 and H3. Consistent with our findings, a previous study of young adults conducted during the COVID-19 pandemic has reported that higher perceived social support from family is associated with higher resilience and lower depression and anxiety scores [24]. Another previous study has reported associations between mental health and social support from family, friends or small groups, communities, organizations or institutions, and society, as well as an association between resilience and mental health among young adults during the COVID-19 pandemic [49]. In addition, this study showed that social support indirectly influenced depression and anxiety through mediation of resilience; therefore, hypothesis H4 was supported. Consistent with our results, previous studies have reported that resilience mediates the association between social support and mental health [33,34].

All indirect effects of fear of infection, difficulties due to social distancing, and anger toward others to depression and anxiety were statistically significant. However, the two paths from difficulties due to social distancing to the two mental health outcomes (depression and anxiety) through social support and resilience were consistent mediations. The remaining mediating paths were inconsistent mediations, which indicated partial support for hypothesis H5. Therefore, this study supports the sequential mediating effects of social support and resilience on the relationship between stress related to difficulties due to social distancing and mental health (depression and anxiety) among young adults during the COVID-19 pandemic. Consistent with these findings, a previous study emphasized social support from family and resilience to COVID-19-related stressors as protective factors for young adults’ mental health [50]. Given these findings, we suggest that interventions to increase social support and enhance resilience can be effective strategies to reduce the risks of depression and anxiety among young adults suffering from stress related to difficulties due to social distancing.

The mediated paths from fear of infection to mental health and from anger toward others to mental health were inconsistent mediations; therefore, we could not determine the mediating effects of social support and resilience in these mediated paths. We confirmed that fear of infection and anger toward others statistically significantly influenced depression and anxiety; however, the underlying mechanisms of the mediating roles of social support and resilience in these relationships were not clear. These findings can be explained by the fact that fear of infection and anger toward others weakly affects depression and anxiety; therefore, the buffering effects of social support and resilience did not appear in these relationships.

### 4.4. Limitations and Strength

This study had the following limitations. First, it used a cross-sectional design; thus, establishing a causal inference was limited. Second, data were collected from panels registered with an online survey institution; therefore, there is a possibility of sampling bias because it is likely that people with greater internet access were more likely to be recruited. Third, self-report measures were used; therefore, respondents were likely to report experiences that were considered socially acceptable and underreport or overreport depending on individual characteristics. Fourth, we provided respondents with only male or female as their gender options in our survey, which might limit our ability to deepen and evolve understanding of gender differences in this phenomenon. Finally, there could have been residual bias due to other unexamined psychological variables that could influence the mediating variable or outcome variables. The influence of other psychological variables not included in this study should be examined in further studies.

Despite the aforementioned limitations, the strength of this study is that it contributes a better understanding of the underlying pathway by which COVID-19-related stress influences depression and anxiety among young adults using large-scale data. Therefore, our findings can inform policymakers and health professionals on how to develop effective interventions to improve mental health among young adults in the context of disasters such as the COVID-19 pandemic.

## 5. Conclusions

This study showed that in the face of the COVID-19 pandemic, the prevalence of young adults suffering from depression and anxiety was high, suggesting that mental health assessment and interventions are urgently needed. In particular, female young adults showed lower resilience and higher depression and anxiety scores than male young adults, indicating they were more vulnerable during this crisis period. The present findings demonstrate that stress from difficulties due to social distancing most greatly influenced depression and anxiety compared with stress from fear of infection or from anger toward others. This indicates that young adults in their most active period were most affected by the stress related to social distancing. Moreover, the findings confirmed the significant mediating roles of social support and resilience in the relationship between stress from difficulties due to social distancing and mental health outcomes (depression and anxiety). Therefore, we suggest that interventions for improving social support and resilience will result in improved mental health outcomes among young adults suffering from stress related to difficulties due to social distancing.

## Figures and Tables

**Table 1:** Social support, resilience, depression, and anxiety according to demographic characteristics (N = 1000).

Variables	n (%) or	Social Support			Resilience			Depression			Anxiety		
	M ± SD	M ± SD	t, F, or r	*p*	M ± SD	t, F, or r	*p*	M ± SD	t, F, or r	*p*	M ± SD	t, F, or r	*p*
Gender													
Male	521 (52.1)	70.03 ± 10.72	−2.10	0.036	62.72 ± 15.90	4.84	<0.001	16.22 ± 17.24	−2.68	0.007	3.87 ± 4.92	−4.44	<0.001
Female	479 (47.9)	71.47 ± 10.95			57.95 ± 15.17			19.09 ± 16.56			5.30 ± 5.28		
Age (years)	21.63 ± 1.69		−0.024	0.449		−0.004	0.903		0.049	0.123		0.033	0.295
Education level													
≤High school graduate ^a^	140 (14.0)	68.52 ± 10.96	3.46	0.032	59.95 ± 18.49	1.61	0.200	19.53 ± 16.92	5.77	0.003	4.94 ± 5.12	4.76	0.009
In college ^b^	647 (64.7)	71.17 ± 10.92		a < b	61.05 ± 15.30			16.27 ± 16.56		b < c	4.20 ± 5.06		b < c
≥College graduate ^c^	213 (21.3)	70.81 ± 10.45			58.88 ± 14.99			20.36 ± 17.82			5.39 ± 5.31		
Religion													
No	702 (70.2)	70.37 ± 10.77	−1.58	0.115	59.47 ± 15.53	−3.00	0.003	17.42 ± 16.78	−0.49	0.626	4.59 ± 5.18	0.29	0.772
Yes	298 (29.8)	71.55 ± 11.01			62.72 ± 16.00			18.00 ± 17.43			4.49 ± 5.07		
Living with spouse													
No	989 (98.9)	70.78 ± 10.86	1.42	0.155	60.40 ± 15.76	−0.76	0.450	17.50 ± 16.94	−1.60	0.110	4.53 ± 5.14	−1.52	0.128
Yes	11 (1.1)	66.09 ± 9.69			64.00 ± 13.49			25.73 ± 17.95			6.91 ± 5.22		
Residential area													
Big city ^a^	537 (53.7)	70.94 ± 11.03	0.48	0.618	61.63 ± 15.68	3.88	0.021	16.92 ± 17.04	0.91	0.402	4.31 ± 5.01	1.39	0.250
Small or medium-sized city ^b^	358 (35.8)	70.67 ± 10.63			58.65 ± 15.77		b < a	18.41 ± 16.39			4.85 ± 5.24		
Rural area ^c^	105 (10.5)	69.81 ± 10.71			60.40 ± 15.40			18.25 ± 18.51			4.85 ± 5.44		
Household economic status													
Low ^a^	316 (31.6)	68.37 ± 11.54	11.50	<0.001	56.37 ± 15.97	19.08	<0.001	19.97 ± 18.01	5.13	0.006	5.11 ± 5.45	3.12	0.044
Middle ^b^	382 (38.2)	71.48 ± 10.42		a < b,c	61.01 ± 15.30		a < b < c	17.12 ± 16.41		c < a	4.46 ± 4.97		c < a
High ^c^	302 (30.2)	72.23 ± 10.26			63.97 ± 15.10			15.72 ± 16.30			4.10 ± 4.99		

In the variable of education level, “^a^” refers to high school graduate or less, “^b^” refers to college student, and “^c^” refers to college graduate or higher. In the variable of residential area, “^a^” refers to big city, “^b^” refers to small or medium-sized city, and “^c^” refers to rural area. In the variable of household economic status, “^a^” refers to low, “^b^” refers to middle, and “^c^” refers to high.

**Table 2:** Levels of COVID-19-related stress, social support, resilience, depression, and anxiety (N = 1000).

Characteristics	M ± SD	Observed Range	Possible Range	Categories	n (%)
COVID-19-related stress	57.33 ± 13.08	0–84	0–84		
Fear of infection	24.15 ± 7.11	0–36	0–36		
Difficulties due to social distancing	14.10 ± 4.84	0–24	0–24		
Anger toward others	19.09 ± 4.31	0–24	0–24		
Social support	70.72 ± 10.85	29–96	24–96		
Resilience	60.43 ± 15.73	0–100	0–100		
Depression	17.59 ± 16.97	0–80	0–80	No depression (0–12)	519 (51.9)
				Depression (13–80)	481 (48.1)
Anxiety	4.56 ± 5.14	0–21	0–21	No anxiety (0–7)	766 (76.6)
				Anxiety (8–21)	234 (23.4)

**Table 3:** Direct, indirect, and total effects of the final modified model.

Path			Direct	Indirect	Total
			β (*p*)	β (*p*)	β (*p*)
Household economic status (low)	→	Social support (SMC = 0.065)	−0.138 (0.001)	-	−0.138 (0.001)
Difficulties due to social distancing	→		−0.178 (0.001)	-	−0.178 (0.001)
Anger toward others	→		0.168 (0.001)	-	0.168 (0.001)
Gender (female)	→	Resilience (SMC = 0.267)	−0.226 (0.001)	-	−0.226 (0.001)
Household economic status (low)	→		−0.109 (0.001)	−0.054 (0.001)	−0.163 (0.001)
Fear of infection	→		0.076 (0.039)	-	0.076 (0.039)
Difficulties due to social distancing	→		0.101 (0.001)	−0.070 (0.001)	0.031 (0.420)
Anger toward others	→		0.133 (0.001)	0.066 (0.001)	0.199 (0.001)
Social support	→		0.393 (0.001)	-	0.393 (0.001)
Gender (female)	→	Depression (SMC = 0.238)	-	0.022 (0.008)	0.022 (0.008)
Household economic status (low)	→		-	0.065 (0.001)	0.065 (0.001)
Fear of infection	→		0.084 (0.015)	−0.007 (0.027)	0.076 (0.028)
Difficulties due to social distancing	→		0.213 (0.001)	0.061 (0.001)	0.274 (0.001)
Anger toward others	→		0.078 (0.020)	−0.079 (0.001)	−0.002 (0.948)
Social support	→		−0.358 (0.001)	−0.038 (0.008)	−0.396 (0.001)
Resilience	→		−0.097 (0.008)	-	−0.097 (0.008)
Gender (female)	→	Anxiety (SMC = 0.261)	0.059 (0.001)	0.019 (0.025)	0.077 (0.001)
Household economic status (low)	→		-	0.060 (0.001)	0.060 (0.001)
Fear of infection	→		0.109 (0.001)	−0.006 (0.039)	0.103 (0.001)
Difficulties due to social distancing	→		0.168 (0.001)	0.057 (0.001)	0.225 (0.001)
Anger toward others	→		0.091 (0.007)	−0.073 (0.001)	0.018 (0.530)
Social support	→		−0.336 (0.001)	−0.033 (0.028)	−0.369 (0.001)
Resilience	→		−0.084 (0.032)	-	−0.084 (0.032)

SMC = squared multiple correlations for structure equations.

## Data Availability

The data presented in this study are available on request from the corresponding author. The data are not publicly available due to restrictions e.g., privacy or ethical.
